# FiatFlux – a software for metabolic flux analysis from ^13^C-glucose experiments

**DOI:** 10.1186/1471-2105-6-209

**Published:** 2005-08-25

**Authors:** Nicola Zamboni, Eliane Fischer, Uwe Sauer

**Affiliations:** 1 Institute of Biotechnology, ETH Zurich, 8093 Zurich, Switzerland

## Abstract

**Background:**

Quantitative knowledge of intracellular fluxes is important for a comprehensive characterization of metabolic networks and their functional operation. In contrast to direct assessment of metabolite concentrations, in vivo metabolite fluxes must be inferred indirectly from measurable quantities in ^13^C experiments. The required experience, the complicated network models, large and heterogeneous data sets, and the time-consuming set-up of highly controlled experimental conditions largely restricted metabolic flux analysis to few expert groups. A conceptual simplification of flux analysis is the analytical determination of metabolic flux ratios exclusively from MS data, which can then be used in a second step to estimate absolute in vivo fluxes.

**Results:**

Here we describe the user-friendly software package FiatFlux that supports flux analysis for non-expert users. In the first module, ratios of converging fluxes are automatically calculated from GC-MS-detected ^13^C-pattern in protein-bound amino acids. Predefined fragmentation patterns are automatically identified and appropriate statistical data treatment is based on the comparison of redundant information in the MS spectra. In the second module, absolute intracellular fluxes may be calculated by a ^13^C-constrained flux balancing procedure that combines experimentally determined fluxes in and out of the cell and the above flux ratios. The software is preconfigured to derive flux ratios and absolute in vivo fluxes from [1-^13^C] and [U-^13^C]glucose experiments and GC-MS analysis of amino acids for a variety of microorganisms.

**Conclusion:**

FiatFlux is an intuitive tool for quantitative investigations of intracellular metabolism by users that are not familiar with numerical methods or isotopic tracer experiments. The aim of this open source software is to enable non-specialists to adapt the software to their specific scientific interests, including other ^13^C-substrates, labeling mixtures, and organisms.

## Background

Genome-wide measurements of cellular mRNA, protein or metabolite concentrations (or their differential concentrations) are current workhorse technologies in functional genomics and systems biology. For a comprehensive analysis of metabolic networks, however, typically also knowledge on the molecular traffic between the metabolites is necessary. These time-dependent in vivo fluxes are the functional complement to the metabolite concentrations, but, in contrast to the concentrations, cannot be detected directly [1]. Instead, intracellular fluxes must be inferred indirectly from measurable quantities, such as nutrient uptake and secretion rates and/or ^13^C-labeling pattern, through methods of metabolic flux analysis [2,3].

To reliably identify a unique distribution of intracellular fluxes, highly controlled culture conditions, extensive physiological, and ^13^C-data are a prerequisite [2]. Although many laboratories have access to the necessary instrumentation, flux analysis remained largely restricted to a handful of expert groups because flux quantification required the simultaneous interpretation of physiological and ^13^C-data. Briefly, complicated isotopomer models of metabolism were used to balance the labeling state of metabolic intermediates or protein-bound amino acids and to identify a best fit of intracellular fluxes to the available data. Several (non-open source) software tools for flux analysis with isotopomer models of varying complexity are available for academic research [4-6], with 13C-FLUX as the probably most advanced one [7]. Furthermore, software tools for automated processing of raw MS [8,9] or NMR data for flux anaylsis are available [10], in the latter case allowing also to calculate flux ratios. Although valuable biological insights can be obtained by isotopomer balancing [11-16], the required expertise in computational analysis and quantitative biology as well as the complexity of the models restricted broader application and wider use as a routine tool.

A conceptual simplification of flux analysis and an appropriate analytical throughput was obtained by splitting the problem in two separate tasks. Firstly, MS-detected ^13^C data are analytical interpreted with probabilistic equations that quantify flux partitioning ratios in so-called metabolic flux ratio analysis [17], akin to an earlier NMR-based approach [18]. In the second step, these flux ratios are used as constraints for a flux balancing calculation in a comparatively simple metabolic network model to estimate absolute intracellular fluxes from the measured extracellular fluxes [19,20]. For non-expert users, the major advantage of this ^13^C-constrained flux balancing is the relative simplicity of the employed models, rapid computation, and a more intuitive data treatment. This also allows to simplify the experimental set-up because the flux ratios are calculated from MS data exclusively. Hence, simple shake flask experiments suffice for standard analyses – although at the cost of flux resolution – thus restricting the use of laborious bioreactor experiments to specific applications. Intuitively, less data suggest less reliable flux estimates, which indeed would be the case if an isotopomer models was used. However, since the flux ratios are analytically determined in a strictly local data interpretation and not in a global fitting procedure, most ratios are from independent measurements and can partly validate each other. For a more comprehensive treatise of flux ratio and net flux analysis please see [3,14,19,21]. Recently, ^13^C-constrained flux balancing was successfully applied to various microorganisms [22-25] and was also the key methodology for higher-throughput flux analyses in our lab [22,26,27].

Based on these conceptual advances, the availability of a user-friendly and robust software for flux analyses becomes the major limitation for wider use. Here we describe the open-source software package FiatFlux that consists of two separate modules for analytical metabolic flux ratio analysis and for ^13^C-constrained flux analysis. FiatFlux condenses our accumulated knowhow and experience on metabolic flux analysis, and was used successfully for teaching and in collaborations with biologically-oriented groups.

## Implementation

We developed the FiatFlux software on a Matlab basis to exploit the Optimization toolbox and the open source environment. FiatFlux consists of two parts with distinct functions: (i) computation of metabolic flux ratios exclusively from MS data in the RATIO module and (ii) estimation of net carbon fluxes within a comprehensive model of metabolite balances from measured extracellular fluxes, previously determined flux ratios, and biomass requirements in the NETTO module. The two modules are run independently, calling either the functions ratio.m or netto.m, respectively.

**Figure 1:**
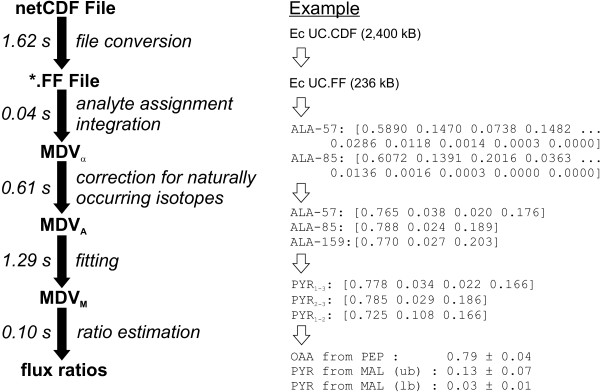
Procedure for derivation of metabolic flux ratios from raw MS data in RATIO (see text for details). For each stage of the analysis, exemplary data and corresponding computation time in seconds are shown on the right and the left, respectively. Times were measured for the analysis of a GC-MS sample on a Pentium 4 1.6 GHz processor.



**Figure 2:**
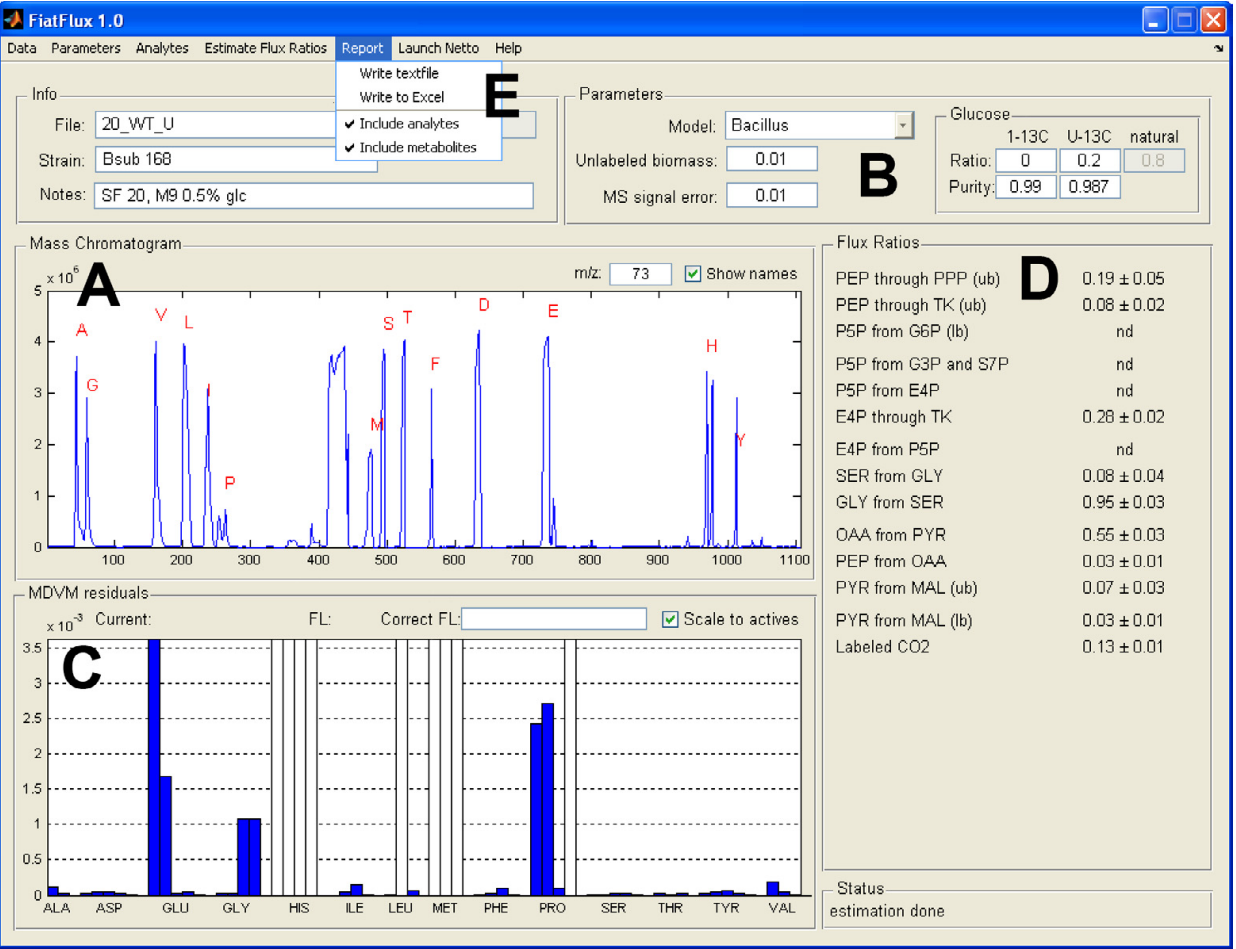
The main window of RATIO. Upon loading of MS data, analytes are first automatically recognized and assigned (A). When necessary, manual assignment of analytes is performed in a different window. The experimental parameters are set by the user (B), then MDV_M _(C) and flux ratios (D) are calculated. Abnormal residuals indicate that the corresponding fragments are outlier, and they can be excluded (white) or reactivated (blue) by a single mouse click on the corresponding bar. Finally, the flux ratios, the MDV_A _and the MDV_M _are exported to a text file or Excel workbook (E).

The RATIO module affords the integration of raw MS data that are passed to the software using the netCDF standard (network Common Data Form) [28] (Figure 1). This format was chosen because it is supported by the proprietary software of most mass spectrometer manufacturers. From a netCDF file, FiatFlux generates a matrix with the total ion counts for each scan (timepoint) and considered m/z value, and searches automatically for known compounds based on their predefined fragmentation pattern. For each recognized analyte, a mass isotopomer distribution vector MDV_α _is extracted from the matrix and normalized such that

where *m*_0 _is the fractional abundance of molecules with monoisotopic mass and *m*_*i*>0 _the abundances of fragments with heavier masses. The mass isotope distribution vector specific to the carbon backbone (MDV_A_) is obtained from MDV_α _upon correction (a) for naturally occurring isotopes of O, N, H, P, S, Si, and C atoms in the derivatization agent [29] and (b) for the presence of unlabeled biomass in the sample, e.g. the inoculum [17]. The MDV_A _are, in turn, used to estimate by least square fitting the mass distribution of their precursors (MDV_M_) in central carbon metabolism [17], along with covariance matrices for each MDV_M_, which are calculated from the experimental error (i. e. comparison of the MDV_A _of fragments with identical carbon skeletons). Faulty MDV_α _measurements are diagnosed by visual inspection of the residuals that result for each MDV_A _in the MDV_M _fitting. In the case of uniformly labeled tracer experiments, diagnosis is based on the fractional labeling of MDV_A _(and MDV_M_) that should equal that of the substrate [30]. Finally, the flux ratios are calculated from the MDV_M _with probabilistic equations [17]. Standard deviations for each flux ratio are calculated using the covariance matrices of MDV_M _by applying the Gaussian law of error propagation [17]. For a more complete treatise of the mathematical/analytical background and the experimental protocols please refer to [30]. User monitoring and intervention is possible at every stage from the graphical user interface (Figure 2).

The set of calculable flux ratios is a function of the biochemical reaction network, the carbon substrates and their corresponding ^13^C-labeling, and the analyte fragments that can be detected by MS. The software is preconfigured to derive metabolic flux ratios for a variety of microorganisms such as yeasts [31,32], *Escherichia coli *[17], *Bacillus subtilis *[23], and others [25] for growth on [1-^13^C]glucose, [U-^13^C]glucose or mixtures thereof. The preconfigured analytes are the TBDMSTFA-derivatized proteinogenic amino acids that are typically detected by robust GC-MS analysis [33]. Notably, FiatFlux is not limited to glucose substrates and can be extended to cope with additional analytes, different derivatization agents or separations, i.e. liquid chromatography or capillary electrophoresis.

**Figure 3:**
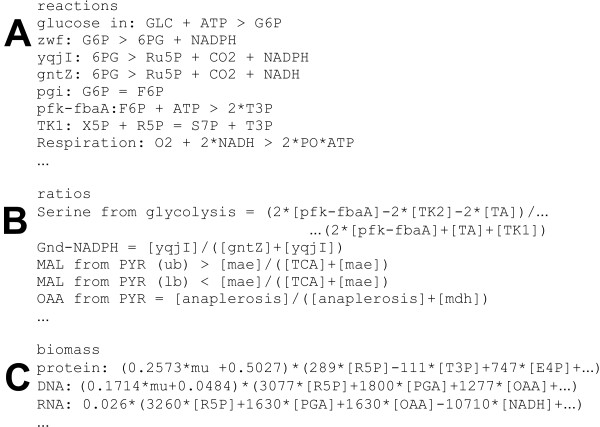
Schematic representation of the analysis workflow in NETTO.

The second module (NETTO) addresses the estimation of absolute in vivo (net) fluxes through a reaction network. This is achieved by global material balances derived from a stoichiometric model and accounting for the withdrawal of precursors during growth (Figure 3). Because of the presence of redundant or interconnected pathways, this system of linear contraints is typically underdetermined [34]. In so-called ^13^*C-constrained flux balancing *[19,20], additional linearly independent constraints are obtained from the experimentally determined flux ratios in the RATIO module that are used to solve the system without further assumptions on energy or redox balances. NETTO features a platform to integrate metabolite balances and ^13^C-derived equality or inequality constraints; i.e. flux ratios that are exactly determined or for which only reaction bounds are available, respectively [19]. Depending on the active set of constraints and reactions, the system may either be underdetermined, determined, or overly constrained. In underdetermined system, NETTO offers either to search within the solution space for the flux distribution that maximizes a particular flux or the product of an intermediate, or estimate all calculable fluxes using the procedure outlined by Klamt et al. [35]. Exactly determined and overly constrained systems are both solved by a least square optimization using Matlab *fmincon *function. This approach permits to simultaneously integrate equality and reaction bound constraints in the calculation, and weight the constraints with the experimental uncertainty [19]. Confidence intervals for each calculated flux are estimated as a function of the experimental errors from the Jacobian matrix of the output function. Inequality constraints (reaction bounds), only contribute to the error criterion if the flux solution would otherwise exceed the upper or lower bounds set by the flux ratio data. This asymmetrical error consideration is described elsewhere [19]. If the boundary constraint is inactive, the confidence interval of the resulting flux (e.g. malic enzyme), is a result of the (stoichiometric) dependence on other fluxes.

**Figure 4:**
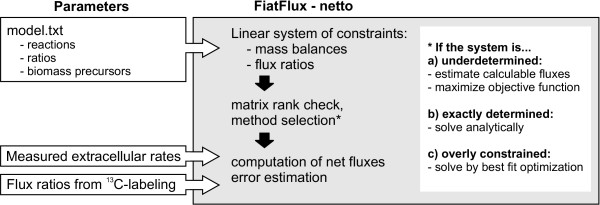
Example of syntax for definition of a model for NETTO. (A) Reactions are described with a unique identifier, educts, products and an operator to set reversibility. (B) Ratios are entered either as equality constraints (=), upper bounds (>), or lover bounds (<), and are defined using the reaction indentifiers. (C) Precursor requirement for biomass formation is expressed with a list of growth-rate dependent withdrawals of metabolites in μmol/gCDW. Separate statements are used for each macromolecular class such as protein, DNA, etc.

**Figure 5:**
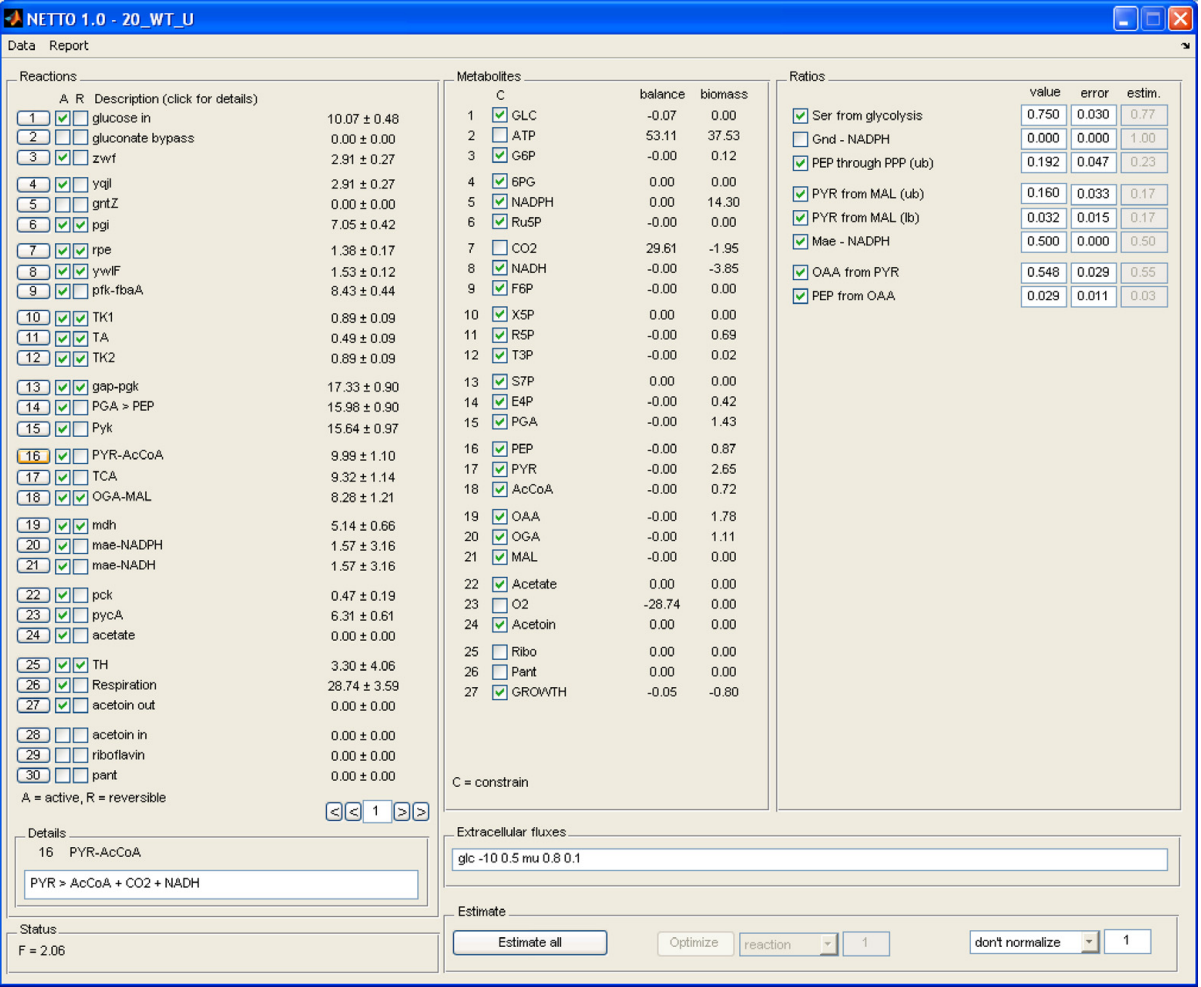
The graphical user interface of NETTO.

In NETTO, metabolic models can be constructed from scratch and error-prone operations such as introduction or modification of reactions are executed by the software. In a text file, the user provides a list of reactions, ratios, and biomass composition with a user-friendly syntax (Figure 4). The information is then automatically translated into properly formatted structures and matrices and saved in a Matlab m-file, that is called by NETTO during computation. The graphical user interface of NETTO permits to freely remove a reaction or modify its reversibility, submit extracellular fluxes or metabolic parameters such as the P/O respiratory coupling, or define which metabolites have to be excluded from balancing, for example oxygen or ATP (Figure 5). Alternatively, default preferences can be defined in the saved model m-file. Whenever a session of RATIO is running in parallel, NETTO imports the value for matching flux ratios.

Both modules offer functions to save all variables and recover work at a later point. Results are visualized directly on the graphical user interface and can be stored to text files or to Microsoft Excel spreadsheets.

## Results and discussion

FiatFlux is the first publicly available software for flux ratio analysis from MS data and, consequently, no comparison can be done with other programs. The scientific value and accuracy of FiatFlux-calculated flux ratios has already been discussed extensively [14,17,25,26,36,37], and consistency between net flux estimates obtained either with ^13^C-constrained flux balancing as in FiatFlux or with global isotopomer balancing was demonstrated previously [19]. Notably, both the calculation of flux ratios from raw MS data in RATIO and the estimation of net fluxes in NETTO is typically completed in a few seconds (Figure 1). This constitutes a major advantage compared to isotopomer balancing, since computation time becomes negligible in relation to the time required by the user to set the experimental parameters. In addition, interpretation of MS data and the integration with measured fluxes are executed independently in FiatFlux. In contrast to methods of isotopomer balancing, this enables the user to discern problems arising from bad measurements or from incomplete metabolic models.

In FiatFlux, user supervision is necessary only when MS-signals are low, saturated, or overlapping. This affects the ion statistics of the corresponding fragment and results in relatively high residuals after inferring MDV_M _from the MDV_A_. Since the residuals are graphically represented on the graphic user interface of RATIO, bad fragments are rapidly identified and excluded with a single click. Also when the quality of the fragments has to be diagnosed in detail, and MDV_M _fitting and flux ratios estimation have therefore to be iterated several times, a correct estimate is obtained within some minutes. Using FiatFlux, a typical user with moderate experience will be able to determine intracellular net fluxes for hundreds of samples per day from previously generated MS data.

The open source nature of FiatFlux, and in particular of the RATIO module, permits to modify and extend its capabilities beyond the predefined features. Although the necessary skills strongly depend on the functionalities to be modified, fundamental biochemical knowledge of the reactions investigated is paramount for every user to understand initial assumptions and critically interpret outcomes. Provided that metabolism of a new organisms to be investigated is similar to that of any of the 4 preconfigured models,, very few adaptations are necessary and the task is manageable by any biochemically-trained biologist. In fact, in previous works we already demonstrated the analysis of about 20 different species with the 4 core models [25,32]. The implementation of new flux ratios or new substrates, however, requires detailed information on mapping of atoms in biochemical pathways, understanding of error propagation, and advanced experience with Matlab syntax, thus is probably limited to experts. Hence, at this stage, we decided to restrict free modification of the preconfigured models by precompiling the corresponding routine. In case a user requires extensions, we encourage to contact the authors to collaborate on a proper integration that ensures correct estimation of metabolic flux ratios and confidence intervals.

Finally, introduction of new GC methods or derivatization procedures is very simple, and can be attained by users with basic familiarity with the Matlab environment. In principle, the same applies to implementing other separation techniques, such liquid-phase systems. Currently, RATIO is not compatible with MS/MS product ion scans.

## Conclusion

FiatFlux condenses the know-how developed over years in our lab and has become our workhorse for quantitative analyses of microbial central carbon metabolism. The software is preconfigured for the most widely used substrate (glucose), the most frequently used (and informative) tracer mixtures, and several model microbes. While this covers about 80% of all current flux applications, it is, of course, not complete. The aim of this open source software is to enable non-specialists to adapt the software to their specific scientific interests, including other substrates and or labeling mixtures. In particular, we aim at biologists that are not familiar with numerical methods or isotopic tracer experiments. In fact, with the availability of this software, the only burden for such studies remains the access to a GC-MS instrument. We hope that this transparent and flexible framework will support further developments.

## Availability

Project name: FiatFlux

Operating system: preferably Microsoft Windows. Some minor problems were encountered using Matlab's graphic user interface with Linux.

Programming language: Matlab R14 (The Mathworks).

Other requirements: Matlab Optimization Toolbox

License: source code is freely available from the authors for academic purposes.

Any restriction to use by non-academics: license required.

## Abbreviations

MDV_α _Mass distribution vector of analyte

MDV_A _Carbon-specific mass distribution vector of analyte

MDV_M _Mass distribution vector of metabolite

TBDMSTFA *N*-(*tert*-butyldimethelsylil)-*N*-methyl-trifluoroacetamide

## Authors' contributions

NZ and EF developed the software. US supported the work. NZ and US wrote the manuscript. All authors read and approved the final version.
